# Audit of Dna/disengagement Guideline in the North Essex Specialist Community Mental Health Team (Scmht), Essex Partnership University Nhs Foundation Trust (Eput)

**DOI:** 10.1192/bjo.2026.11673

**Published:** 2026-06-30

**Authors:** Rupam Kumari, Basavaraja Papanna

**Affiliations:** EPUT, Colchester, United Kingdom

## Abstract

**Aims:**

Research has shown that inadequate follow-up after a DNA incident significantly contributes to Patient Safety Incidents, Domestic Homicide Reviews, and Safeguarding Adults Reviews. Furthermore, this disengagement not only threatens patient safety but can also lead to increased hospital admissions and strain the NHS’s financial resources.

In light of these substantial risks, we undertook our first audit in 2023 to evaluate our adherence to the relevant policies. To ensure we make meaningful improvements, we conducted a reaudit in 2025, focusing on the North Essex Community Mental Health Team’s compliance with DNA and disengagement guidelines.

EPUT’s Guideline Implementation: April 2018, Amended August 2022:

Document actions based on risks after DNA.

- Liaise with service user/family/friend/carer, GP and other agencies involved in care.
- offer another appointment.
- Review the care and treatment plan, including the crisis and contingency plan.
- Consider a home visit (Cold call), refer to the AMHP service for a Mental Health Assessment or refer for a welfare check if there is an imminent threat to life (patient or someone else).
- Discuss within the MDT meeting/Line manager.
- Call a Professional meeting or Multi-agency meeting as required, indicated by the risk.
- Discharge may be considered if the service user has been made aware that this action will be taken if they do not attend another appointment and there is no risk to themselves and/or others.

**Methods:**

A retrospective analysis of outpatient follow-ups from January to June 2025. Our sample included 137 service users who did not attend appointments with various professionals, including doctors, nurses, and psychologists.

Following the first audit cycle, More Staff were aware of the existing trust guideline and the importance of documenting the action plan.

**Results:**

**The outcome of the reaudit compared to the data from the first audit:**

* **Positive Changes Compared to Previous Audit:**

- Documenting Risk: 95% (↑ from 48.2%)

- Risk Management Plans: 58% (↑ from 49.4%)

- Notifying Involved Professionals or MDT: 60% (↑ from 50.5%)

- Patients Offered Another Appointment: 92% (↑ from 77.6%)

*Areas of Concern:

- Confirming Appointments: 92% (↓ from 94.1%)

- Contacting Patients after DNA: 74% (↓ from 81.1%)

**Conclusion:**

While we are making significant strides in adhering to trust policies, there is still room for improvement–particularly in the documentation process. Enhancing this aspect will provide clearer evidence of our compliance with disengagement guidelines. Therefore, the disengagement care plan and support decision tool for disengagement are recommended. Our commitment to continuous improvement leads to the determination to conduct future audits to ensure ongoing progress and accountability.

